# Rabies control in high-burden countries: role of universal pre-exposure immunization

**DOI:** 10.1016/j.lansea.2023.100258

**Published:** 2023-08-01

**Authors:** Lonika Lodha, Ashwini Manoor Ananda, Reeta S. Mani

**Affiliations:** Department of Neurovirology, WHO Collaborating Centre for Reference and Research in Rabies, National Institute of Mental Health and Neurosciences (NIMHANS), Hosur Road, Bengaluru, Karnataka 560029, India

**Keywords:** Rabies, Immunization, Zoonosis

## Abstract

Rabies is a fatal zoonotic encephalitis that is responsible for approximately 59,000 deaths worldwide every year. A significant portion of these deaths, about one-third, occur in India alone. In order to meet the World Health Organization's objective of eliminating dog-mediated rabies by 2030, India has made considerable progress in this regard. However, implementing the current strategies of canine immunization, sterilization, and providing post-exposure prophylaxis to exposed individuals is challenging in a large and diverse country like India. This article aims to highlight the limitations of relying solely on post-exposure prophylaxis for the prevention of human rabies. Moreover, it presents the necessity and rationale for including pre-exposure immunization in India's national immunization schedule.

## Introduction

Rabies is zoonotic encephalitis caused by the rabies virus (RABV) and other viruses belonging to the Lyssavirus family. The infection is nearly always fatal, resulting in approximately 59,000 deaths worldwide each year.^1^ The burden of this disease is predominantly carried by underserved and resource-limited regions in Asia and Africa, with children under the age of 15 accounting for 40% of reported deaths. Rabies is prevalent in over 150 countries worldwide, and India alone accounts for one-third of the global rabies cases, resulting in approximately 20,000 deaths annually.^1^ These figures are likely underestimated due to insufficient surveillance and reporting. Consequently, effective rabies control measures in India are crucial for attaining the global goal of eliminating rabies.

Aligned with the global objective of eliminating human deaths caused by dog mediated rabies by 2030 (Zero by 30), India introduced its National Action Plan for the Elimination of dog-mediated Rabies (NAPRE) in 2021.^2^ The government has issued a directive for human rabies to be made a notifiable disease in all states, which is a crucial milestone in establishing effective reporting and surveillance systems. Subsequently, it will enhance the availability of diagnostic services and vaccines while allowing for the adaptation of rabies control policies to identify and address any existing gaps.

While rabies is fatal once clinical symptoms manifest, it is preventable through either pre-exposure or post-exposure prophylactic immunization (PEP and PrEP, respectively). The WHO recommends the use of purified cell culture and embryonated egg-based rabies vaccines (CCEEVs), which can be administered intramuscularly or intradermally following approved regimens.

The primary objective of PEP is to eliminate or neutralize the RABV at the bite site, thereby preventing its entry into the nervous system. PEP vaccination should be initiated as soon as possible following exposure and should be accompanied by appropriate wound care and rabies immunoglobulin (RIg) infiltration in some cases. On the other hand, the goal of PrEP is to stimulate the immune system to produce RABV neutralizing antibodies (RVNA) prior to any potential exposure. If subsequent exposures occur in these individuals, only a few vaccine boosters are needed to trigger an anamnestic response, resulting in a rapid increase in RVNA levels to prevent infection.

It is important to note that pre-exposure rabies vaccination is not currently included in India's national immunization schedule. The purpose of this viewpoint article is to shed light on the challenges and drawbacks of the current rabies vaccination policy in India and advocate for the inclusion of PrEP in India's national immunization schedule, as well as in other countries burdened with a high incidence of rabies.

## Current strategies for rabies control in India

Dogs serve as the main reservoir hosts for RABV, being responsible for transmitting the infection in the majority of human cases worldwide. Therefore, effective control of rabies in dogs is essential for achieving a long-term and sustainable reduction in human rabies cases, which can be accomplished through mass dog vaccination and population control measures. An excellent example of the successful elimination of human rabies was seen in the state of Goa, India, through a combination of strategies, including access to PEP, conducting rabies awareness campaigns, and implementing enhanced surveillance activities.^3^ Stray dog vaccination and population control measures have shown promising results in specific regions like Jaipur city, the Nilgiris district of Tamil Nadu, and the state of Sikkim.^2^

However, expanding these successful interventions nationally faces significant challenges due to the large and inaccessible population of unvaccinated stray dogs. Canine vaccination programs are estimated to cost three to ten times more than human vaccination programs, and their effectiveness relies on combining them with a canine population control program.^4^ Achieving success at a larger scale requires a sustained political commitment and substantial financial resources.

The advantages and limitations of various rabies control strategies are listed in Table 1. Currently, the primary strategy employed on a national scale to prevent human rabies is the administration of PEP to individuals with suspected rabid exposures.

**Table 1:** Advantages and limitations of current rabies control strategies.

Strategy	Advantages	Limitations
Mass dog vaccination	Most cost-effective method of rabies control Only method that targets viral reservoir Successfully demonstrated to be an effective control measure in several countries	Minimum 70% coverage requirement Inaccessible free-roaming dog population Does not prevent wildlife-derived rabies
Post exposure prophylaxis	Excellent safety profile Almost 100% effective Reduced doses with intradermal route	Lack of accessibility Need of prompt administration Cost of RIG Non-compliance due to long schedule
Raising public awareness	Enhances implementation of other control strategies Increased appreciation of public health impact leads to prioritisation by policy-makers	Strong cultural and traditional misconceptions in the community Language and communication barriers

## Rabies post-exposure prophylaxis: challenges

Timely and affordable delivery of adequate PEP is crucial to prevent clinical rabies after animal exposures. However, in a large and diverse country like India, achieving this goal can be challenging.

### High incidence of animal exposures

India has a significant population of stray or free-roaming dogs, estimated at 60 million,^5^ which contributes to approximately 17.4 million animal bite incidents annually.^6^ Among patients seeking healthcare after animal exposure, a high proportion, up to 91.0%, experience category III exposures.^7^ Children are particularly vulnerable as they often experience category III exposures and may require both RIg and vaccination.^8^ Unfortunately, children often hesitate to report dog bites to their parents due to fear of punishment, resulting in a potential lack of PEP administration.

### Lack of awareness among general public and HCWs

Multiple studies have shown a concerning lack of awareness about rabies prevention, not only among the general public but even among healthcare workers (HCWs). For instance, in a survey, only 60.4% of the participants had prior knowledge of rabies, and only 22.9% were aware of the importance of wound washing after a dog bite. Moreover, only 63.6% of the participants expressed a willingness to visit a healthcare facility or seek PEP after being bitten by an unknown dog.^9^

Due to lack of knowledge, individuals often resort to using ineffective and potentially harmful remedies instead of seeking proper PEP after animal bites. This includes using irritant household remedies, consulting faith healers and quacks, or using household preparations like chilli, lime, kerosene, turmeric, herbal medicines, and homemade oils for wound treatment. Shockingly, studies have shown that a significant percentage of animal bite victims, ranging from 64.2% to 80%, rely on these inappropriate practices.^10^

In a survey involving 96 HCWs, it was found that only 13.6% of them considered licks over broken skin as a category III exposure. Additionally, less than half of the respondents were aware of the minimum duration for wound washing (10–15 min), and less than one-third knew that the full dose of RIg should be administered in and around the wound site.^11^

### Lack of availability and affordability of post-exposure prophylaxis

Accessible, affordable, and well-equipped anti-rabies clinics (ARCs) are essential for effective prevention of rabies in animal bite victims. However, in a large and geographically diverse country like India, ensuring uninterrupted supply and stock of rabies vaccines and immunoglobulins presents significant challenges. A nationwide assessment of ARCs revealed that only 54.3% of the surveyed ARCs had wound washing facilities. Vaccine stock-outs were reported in 18.4% of the ARCs, and only 54.3% of the clinics provided RIg administration.^12^ Disparities in the availability of RIg among different states of the country, along with a lack of monitoring and reporting of immunoglobulin usage, have also been observed.^13^

While government ARCs provide free PEP, patients may face challenges due to distance or time constraints. Private sector ARCs are often unaffordable for many patients, particularly when RIg is required. In addition to the direct costs of vaccines and biologicals, the indirect costs such as loss of wages and travel expenses pose further challenges for patients, and contribute to delays in initiating PEP, which reduces its effectiveness.^14^

### Non-compliance to post-exposure prophylaxis

Poor compliance to PEP is a significant factor contributing to rabies deaths, despite adequate availability. Compliance to PEP varies depending on factors such as location (rural or urban), route of administration (intramuscular or intradermal), and dosage schedule. Studies have reported variable compliance rates for different PEP regimens. For example, compliance with the four-dose intradermal regimen can be as low as 52·3%.^15^ The implementation of an integrated bite case management system with digital reminders to patients can improve PEP compliance,^3^ but it may not be feasible in developing nations due to financial and logistical constraints.

Compliance to receiving RIg, which is necessary for all category III exposures, is even lower than vaccine compliance. In various studies, it has been found that less than half of the patients who require RIg actually receive it,^15^ due to unavailability of RIg or the unwillingness of patients to receive the treatment. Proper administration of RIg, which involves infiltration in and around all the wounds,^1^ can be challenging, especially for children or patients with multiple wounds.

### PEP failure

Reported cases of PEP failure are often not due to true vaccine failure but rather deviations from the WHO-recommended PEP guidelines, such as omission or improper administration of RIg, lack of primary wound care, inadequate vaccine doses, inappropriate site of vaccination etc.

True PEP failures are usually linked to bites in highly innervated regions like the face, neck, arms, fingers, or multiple bites. Direct virus inoculation into a nerve results in a shorter incubation period, compromising the efficacy of active and passive immunization.^1^

### Psychological and mental health impact of animal bite

In cases of animal bites, despite adequate PEP, the fear and anxiety associated with the possibility of contracting a fatal illness like rabies can be distressing for the victim and their families, specially in remote or low-resource areas where access to post-exposure prophylaxis (PEP) may be delayed. The waiting period until receiving PEP can be a time of heightened stress and anxiety, as individuals worry about their health and the potential consequences of the bite.

## Current recommendations for pre-exposure prophylaxis of rabies

The latest WHO Position Paper on Rabies Vaccines^16^ recommends PrEP only for a subset of the population at high risk of rabies exposure. This includes individuals in highly endemic areas or areas without access to timely and adequate PEP, owing to the high risk of exposure to rabies due to their occupation or recreational activities. PrEP is also recommended for travellers from non-endemic to endemic areas, and in areas where the incidence of dog bites exceeds 5% per year or where vampire bat rabies is prevalent. The current recommendations in India align with these guidelines. The Indian Academy of Pediatrics suggests the rabies vaccine as an optional immunization for children at a high risk.^17^

The large-scale implementation of PrEP is not currently considered cost-effective by the WHO unless the incidence rate of dog bites exceeds 5%. However, the cost of childhood PrEP becomes equal to PEP in a setting of high incidence of dog bites and prudent choice of vaccination regimens.^18^ A study from Philippines has showed that universal PrEP programme for children would be cost-effective in the long term, based on a static decision tree model.^19^ Studies from India have reported PrEP to be highly cost effective in children, with ID as well as IM routes,^20^ and that it is operationally feasible to implement in areas with animal bite rates of more than 5%.^1^

### Effectiveness of PrEP in prevention of rabies

There is abundant evidence supporting the safety and immunogenicity of pre-exposure vaccination for rabies in children. In contrast to the several deaths reported despite PEP, PrEP failure has never been documented, except a single case in 1983, which was attributed possibly to concurrent chloroquine administration.^21^ Pre-exposure prophylaxis for rabies provides long-lasting protection and is capable of eliciting an adequate immunological response even with a single booster dose for several years after the initial vaccination.^22^

RIg is mandatory and lifesaving in individuals with Category III exposures. However, using RIg has various challenges, including availability, cost, and proper administration. If a previously immunized person (PrEP) experiences a rabid exposure, RIg is unnecessary, and the vaccination schedule can be shortened. Additionally, for previously vaccinated individuals, post-exposure vaccination is not an urgent life-saving measure, allowing for some delay if PEP is not immediately accessible.

While the current WHO recommendation for PrEP is a two-visit intradermal or intramuscular regimen, there is evidence that a single-visit intramuscular or intradermal vaccine provides similar protection.^23^ Intradermal vaccination, a cost and dose-saving alternative to intramuscular vaccination, is already being used for PEP in several public sector ARCs in various Indian states. Implementing such approaches could potentially serve as a simple and inexpensive means of incorporating routine rabies vaccination into the national immunization program.

### Challenges in administration of PrEP

As discussed in the preceding sections, relying solely on PEP for controlling human rabies has drawbacks, which can be overcome by implementing PrEP. However, this introduces new challenges, particularly regarding the cost and logistics of such a program. Allocating existing vaccine stocks for PrEP raises concerns about shortages for PEP, a critical intervention. These challenges can be addressed by developing a clear vaccination strategy, expanding vaccine production, and creating a dedicated PrEP stockpile.

ID vaccination is dose- and cost-sparing, but only if the required number of individuals are vaccinated at a time to utilize the entire vial. Successful administration of ID vaccines may also require some skill and training for the healthcare workers.

For successful implementation of a large-scale rabies PrEP program, establishing a strong, sustainable funding pipeline is crucial. This can only be achieved through significant policy changes that integrate rabies control into existing national health programs. Additionally, public awareness campaigns are essential to promote vaccine acceptance and uptake while also generating political determination to combat rabies.

### Scope for co-administration or multivalent administration of rabies vaccine

It is well-documented that the rabies vaccine can be administered together with other routine childhood vaccines, such as diphtheria, tetanus, pertussis, polio, and Japanese encephalitis.^24^ Various bivalent and multivalent rabies vaccine preparations have been developed and studied, offering an economically and logistically feasible approach for large-scale public health programs. Research has shown successful inclusion of rabies vaccination in routine immunization programs,^25^^,^^26^ providing valuable examples to guide future decisions in rabies-endemic countries like India. Clinical trials across Asia have extensively evaluated the safety and immunogenicity of intradermal PrEP for children, demonstrating its effectiveness in providing adequate protection.^27^, ^28^, ^29^

## Conclusion

The Universal Immunization Programme in India already provides nationwide coverage for nine infectious diseases and extends sub-national coverage for three additional infections. India's National Family Health Survey reports that 83.8% of children aged 12–23 months are fully vaccinated, highlighting the existing infrastructure for vaccine delivery. Leveraging this robust system, India can incorporate large-scale pre-exposure rabies vaccination into its national immunization schedule, building on successful milestones in immunization, such as the elimination of polio (2014) and maternal/neonatal tetanus (2015). This practical approach strengthens efforts to prevent rabies-related deaths and utilizes established resources for the benefit of the population.

Protecting vulnerable populations in rabies-endemic areas is a moral and ethical responsibility. It is concerning that permanent residents of these areas are not provided with the same preventive measures as travellers, despite facing similar or higher risks of rabies exposure. Addressing this disparity is crucial for equitable access to rabies prevention. By including rabies PrEP in the national immunization schedule for residents in these areas, authorities can ensure consistent protection and prioritize the health and well-being of those most susceptible to rabies infection in these regions.^30^

## Contributors

RSM conceptualized the manuscript and contributed to manuscript editing and finalisation. LL principally drafted and visualized the manuscript. AMA contributed to manuscript development, editing, and finalisation. All authors have read and approved the final version of the manuscript. All authors had final responsibility for the decision to submit for publication.

## Declaration of interests

We declare no competing interests.
